# *Enterovirus D*: A Small but Versatile Species

**DOI:** 10.3390/microorganisms9081758

**Published:** 2021-08-17

**Authors:** Ines Cordeiro Filipe, Mariana Soares Guedes, Evgeny M. Zdobnov, Caroline Tapparel

**Affiliations:** 1 Department of Microbiology and Molecular Medicine, University of Geneva, 1206 Geneva, Switzerland; Mariana.darochasoaresguedes@unige.ch; 2 Department of Genetic Medicine and Development, Switzerland and Swiss Institute of Bioinformatics, University of Geneva, 1206 Geneva, Switzerland; Evgeny.Zdobnov@unige.ch

**Keywords:** *Enterovirus*, *Enterovirus D*, EV-D68, EV-D70, EV-D94, EV-D111, EV-D120, *Picornavirus*

## Abstract

Enteroviruses (EVs) from the D species are the causative agents of a diverse range of infectious diseases in spite of comprising only five known members. This small clade has a diverse host range and tissue tropism. It contains types infecting non-human primates and/or humans, and for the latter, they preferentially infect the eye, respiratory tract, gastrointestinal tract, and nervous system. Although several *Enterovirus D* members, in particular EV-D68, have been associated with neurological complications, including acute myelitis, there is currently no effective treatment or vaccine against any of them. This review highlights the peculiarities of this viral species, focusing on genome organization, functional elements, receptor usage, and pathogenesis.

## 1. Introduction

Enteroviruses (EVs) are among the most prevalent viruses worldwide and several of them are important human pathogens. This group of viruses is characterized by a high genetic and phenotypic diversity, although the determinants behind it are still poorly understood. The *Enterovirus D* species includes five members with distinct symptomatology, illustrating EV diversity. This article reviews the current knowledge on *Enterovirus D* species. Recent findings on the members of this species are described, as well as the crucial role played by EV-D and more generally by EV non-structural proteins in the pathogenesis of these viruses.

## 2. *Enterovirus D* Classification

*Enterovirus* genus includes 15 different species, each further subdivided in numerous types. Seven species, *Enterovirus A* to *D* and *Rhinovirus A* to *C*, contain human-infecting viruses [1,2,3], while the remaining eight species contain viruses with a wide host range from camelids to rodents and even marine mammals [4].

*Enterovirus D* is among the smallest species of the *Enterovirus* genus, counting only five types, but the host range, tissue tropism, and associated diseases are very diverse. EV-D68 and EV-D70, the first discovered EV-Ds, infect only humans but, unlike most EVs from the *A* to *C* species, they are not considered enterotropic viruses and are rather associated with respiratory and eye infections, respectively [5,6]. Although the EV-D68 genome has been detected in stools samples, no infectious viruses were isolated [7]. EV-D68 is also the only acid-sensitive in the *Enterovirus D* species [8,9]. The other three EV-D types, EV-D94, EV-D111, and EV-D120, were detected in Africa and remain poorly characterized. EV-D94 was discovered in 2007 in samples from sewage in Egypt and from acute flaccid paralysis (AFP) cases in the Democratic Republic of the Congo [10]. EV-D111 was identified both in human and primates stool samples, while EV-D120 was only detected in stool samples of wild non-human primates [11,12]. Important to note, the circulation of these viruses in humans may be underestimated as exemplified by seroprevalence studies highlighting the high prevalence of anti-EV-D94 antibodies in the Finnish population [10].

**Figure 1:**
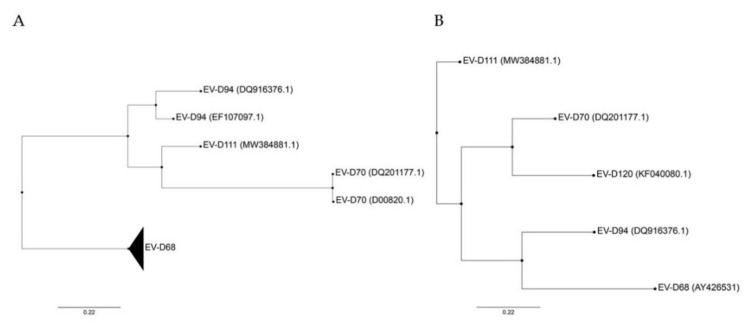
Phylogenetic trees constructed from complete genomes (**A**) and VP1 (**B**) of members of the Enterovirus D species. Both trees were rooted on porcine enterovirus 8 (AF406813), not shown. Only VP1 sequence was available for EV-D120, excluding this genotype from (**A**). Phylogenetic trees were constructed with PhyML using the Smart Model Selection, based on ungapped multiple sequence alignments produced with Muscle [14,15,16,17]. GenBank accession number are detailed in parentheses for each virus except for EV-D68 that includes several strains (AY426531, AB601882.2, AB601883.2, JX070222.1, JX10184.1, KF726085, KM851231.1, KM892500.1, KP240936, KP745755.1, KP745766.1, KP745767.1, KT285319.1, MK105982.1, MN240505, MN245981).

Based on the full genome sequence, EV-D94 is more closely related to EV-D111 and EV-D70 than to EV-D68 (Figure 1A). In fact, it has been proposed that EV-D94 and EV-D111 evolved by intertypic recombination, whereas no evidence has been found to support such recombination between these two viruses and EV-D68 [11]. In contrast, based on VP1 sequences, EV-D94 is closely related to EV-D68 and EV-D70 to EV-D120, while EV-D111 appears to be the most distinct member of the species (Figure 1B). This variation in VP1 may reflect different selective pressures. Important to note, the VP1 sequence divergence does not account for the observed intraspecies phenotypic diversity and is not specific to *Enterovirus D*. Indeed, other EV species infecting humans also contain viruses capable of causing a wide range of diseases (reviewed in Reference [3]) with the exception of the three rhinovirus (RV) species that contain only viruses causing respiratory infections. Even though recombination has been determined as a crucial mechanism for EV evolution, it seems to be a rare event among human-infecting EV-Ds probably due to distinct in vivo tropism [11,13].

## 3. *Enterovirus D* Genome Organization and Functional Elements

The EV genome is a positive strand RNA molecule of 7100 to 7450 nucleotides (nt) [2,3]. It contains a single open reading frame (ORF) encoding a polyprotein that, after processing, gives rise to 11 mature proteins. The ORF is divided into three main regions: P1 that includes the coding sequence for the four structural proteins (VP1–VP4) and both P2 and P3 that encode the seven non-structural proteins (2A–2C and 3A–3D). A recent study highlighted that some enterotropic EVs harbour an upstream ORF that improves replication in human intestinal organoids, contesting the old dogma of EVs expressing a single polyprotein [18]. However, this upstream ORF is absent in the *Enterovirus D* species even for the enteric members [18].

### Cis-Acting RNA Structures

**Figure 2:**
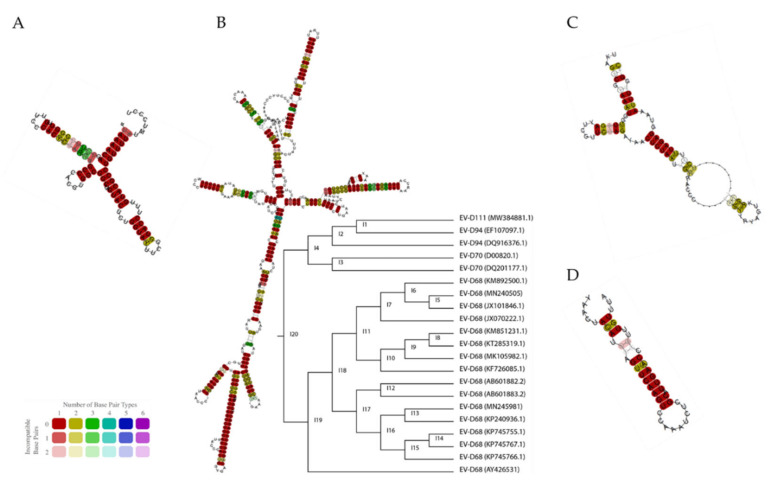
Structure of the cis-acting elements present in EV-D genomes: 5′ CL (**A**), 5′ IRES with its respective topology tree (**B**), 3′ UTR (**C**), and cre (**D**). Contemporary EV-D68, EV-D68 Fermon, EV-D94, EV-D70, and EV-D111 were included. The RNA fragments were extracted using the AliView [17] sequence alignment editor and then were simultaneously folded and aligned using LocARNA from Freiburg RNA tools. The sequence alignment of the different regions is available in Supplementary Figure S1.

Two untranslated regions (UTR), which contain several structural elements crucial for the viral life cycle, are flanking the EV coding sequence. The 5′ UTR contains a 5′ cloverleaf (5′-CL) (Figure 2A) and an internal ribosome entry site (IRES) (Figure 2B), while the 3′ UTR presents repeated stem-loop structures (Figure 2C). The 5′-CL provides a platform for the assembly of ribonucleoprotein (RNP) complexes composed of viral and host proteins. These RNP complexes interact with the 3′ UTR, allowing for genome circularization, a prerequisite for viral replication [19,20,21,22]. Downstream to the 5′-CL, the type I IRES allows for the cap-independent translation of the polyprotein [21]. Translation initiation depends on IRES recognition by specific cellular proteins such as eIF3, eIF4A, and eIF4G, allowing for the recruitment of the 40S ribosomal subunit in the absence of a 5′ cap [21,23,24]. The 3′UTR also contains elements important for viral replication such as the oriR. The OriR is composed of several stem-loops that promote the assembly of RNP complexes, which, as mentioned before, contribute to genome circularization and RNA synthesis [22,25,26]. The 3′UTR ends with a poly(A) tail that also participates in genome circularization and the initiation of viral RNA synthesis [27,28]. Finally, an internal hairpin structure named cis-acting replication element (*cre*), located in the coding sequence (in 2C for *Enterovirus A* to *D* species), is essential for genome replication by serving as a template for VPg uridylation (VPgpUpU) and creating a primer for RNA synthesis [27,29]. The *cre* is highly conserved among EV-D members (Figure 2D).

As the 5′ and 3′ UTR define replication and translation efficiencies, they represent important virulence determinants. Several studies have demonstrated poliovirus (PV)dependence on the 5′ UTR for its neurovirulence [30,31,32,33]. The UTRs of EV-D members are well conserved, particularly the 5′ CL (Figure 2A), even if some variations are observed for the 5′ IRES and 3′UTR (Figure 2B,C). The topology of the phylogenetic tree of the 5′ IRES, considering both sequence and structure similarity, matches the whole genome tree (Figure 1A) with EV-D94, EV-D70, and EV-D111 clustered together, while EV-D68 strains are grouped in a different cluster. Interestingly, within this EV-D68 cluster, the EV-D68 Fermon reference strain (AY426531) presents a distantly related IRES (Figure 2B). There is increasing evidence that the IRES structure modulates EV-D68 infection severity and the mutations affecting the RNA folding have been identified in recent strains [23]. These mutations lead to higher IRES activity in neuronal and lung cells [23]. It is therefore possible that, like for PV, recently acquired mutations in the 5′ UTR of EV-D68 contribute to an increased respiratory/neuronal virulence [23]. The 3′ UTR may also represent a virulence factor. Stress granule (SG) proteins have been shown to interact with the 3′ UTR of EV-D68. A recent study suggested that SG can chelate the viral RNA, inhibiting viral transcription and translation [34]. However, EV-D68 is able to antagonize SG antiviral activity [34]. Similarly, other EVs have been reported to interact with SG such as EV-A71 and PV [35,36]. Thus, changes in the 3′ UTR structure may modulate the viral response to SG. Further studies are necessary to support this hypothesis.

## 4. Structural and Non-Structural Proteins

The four structural and the seven non-structural EV proteins are generated by the co and post-translational cleavage of the viral polyprotein by the viral 2A and 3C proteases. Important to note, three uncleaved protein precursors, namely 2BC, 3AB, and 3CD, present specific functions increasing the coding capacity of the small EV genome. Most data about the functions of EV structural and non-structural proteins derive from studies performed with PV, coxsackievirus B3 (CVB3), and EV-A71, while only few investigations have been conducted on EV-Ds. The information presented in the next section is thus mainly based on these model viruses.

### 4.1. Structural Proteins

The P1 region includes four structural proteins, VP1 to VP4, that form the viral icosahedral capsid. Initially, VP0 (the precursor of VP4 and VP2), VP1, and VP3 assemble to form provirions that become mature only after the RNA-induced processing of VP0 into VP2 and VP4 [37]. The icosahedral arrangement is built with 60 repeating protomers, each containing the four structural proteins, VP1, VP2, VP3, and VP4. While VP1, VP2, and VP3 are located at the capsid surface, VP4 is found in the internal side of the virion and is linked to a saturated fatty acid by a myristoylation process that participates in viral entry and assembly [37]. Across the capsid surface, EVs present deep circular depressions termed canyons, which commonly serve as receptor binding site. A pocket factor, frequently a lipid moiety, can be found at the floor of the canyon (except for *Rhinovirus C*) and contributes to particle stability [37,38].

In addition to their role in entry and viral assembly, structural proteins are involved in other steps of the viral life cycle. It has recently been shown that CVB3 VP1 translocates to the nucleus and modulates cell cycle progression, thereby promoting viral replication. By shortening the G2-M phase and prolonging S phase, viral translation and the production of virions are enhanced [39,40]. These findings propose a novel EV pathogenic mechanism which illustrates that structural proteins may have functions beyond their structural role.

### 4.2. Non-Structural Proteins

P2 and P3 comprise all the necessary proteins for viral replication, namely 2A–2C and 3A–3D, respectively. In general, P2 proteins ensure viral replication by interacting with the host cell, while P3 proteins are actively involved in genome replication. In spite of this, EV non-structural proteins are multifunctional with overlapping functions that can act at several steps of both the viral and cell cycle.

#### 4.2.1. The Viral Proteases: 2A and 3C

**Figure 3:**
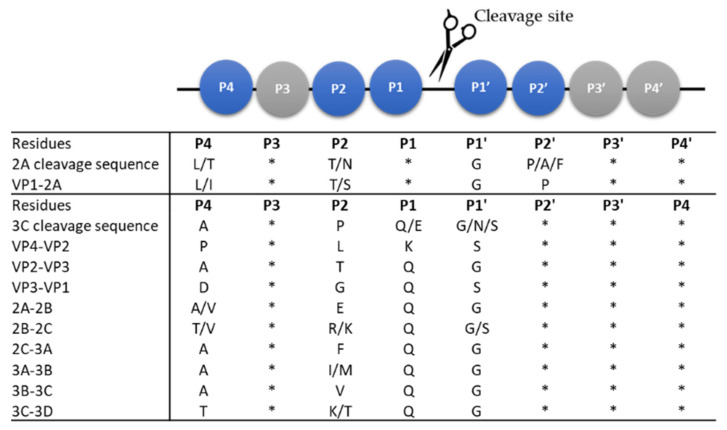
*Enterovirus D* cleavage recognition sequences of the 2A and 3C proteases. Comparison between the EV 2A and 3C cleavage sequence (in the table as “2A cleavage sequence” and “3C cleavage sequence”, mentioned in the text and reviewed in Reference [41]) and the sequence observed in EV-D members at the different cleavage sites across the polyprotein. Based on the sequences of EV-D68 (AY426531), EV-D94 (EF107097 and DQ916376), EV-D70 (DQ201177 and D00820), and EV-D111 (MW384881). The critical positions for recognition by the viral proteases are in blue (residues listed in the table), while the less conserved positions are in grey (* undefined residues).

EVs encode two cysteine proteases, namely 2A and 3C, with similar tertiary structures (reviewed in References [41,42]). In terms of sequence identity, 2A and 3C can be quite divergent across the species, although the catalytic site is fully conserved across EVs [41]. This suggests a conserved sequence specificity and cleavage site [41]. P4, P2, P1, P1′, and P2′ (according to the Schechter and Berger nomenclature) were identified as the most important residues for EV proteases cleavage (Figure 3) [41,43,44]. In particular, P1′ is critical, 2A recognizes only a glycine at this position, and 3C recognizes glycine, asparagine, or serine (Figure 3) [41,44]. The remaining residues for 2A cleavage are, in order of importance, a threonine or an asparagine at P2; a proline, an alanine, or a phenylalanine at P2′; and a leucine or threonine at P4. Concerning 3C, it preferentially recognizes a glutamine or glutamate at P1, and an alanine and a proline at positions P4 and P2, respectively [41]. Important to note, these residues can vary, as observed for the *Enterovirus D* species (Figure 3).

Upon translation, EVs depend on the proteolytic processing of the long polyprotein to release the mature viral proteins and successfully replicate. Indeed, the very first task of 2A after its translation is self-cleavage between its N-terminus and C-terminus of the VP1, separating itself from the precursor P1, while the polyprotein continues to be translated [42]. This is the only cleavage of the viral polyprotein made by 2A, while 3C and even its precursor 3CD are responsible for all other cleavages, with the exception of VP0, presumably cleaved via an autocatalytic process involving RNA [42,45].

2A, 3C, and even the precursor 3CD do much more than polyprotein processing. In fact, they orchestrate several strategies to favour viral replication. A well-known EV strategy is the shutoff of the host translation by the 2A cleavage of eIF4G [46]. eIF4G is a translation initiation factor responsible for recruiting ribosomes to the cap structure of cellular mRNAs. 2A cleaves eIF4G into 2 domains without impairing EV translation because the C-terminal domain is sufficient to attach viral RNA to the ribosome and initiate translation [21,23,24,47,48,49]. Thus, the eIF4G cleavage will only impair the host cap-dependent translation, promoting translation of viral transcripts via their IRES [41,46]. 2A of several EVs (PV, CVB3, and RV-A16) also cleaves nucleoporins, particularly Nup153, Nup98, and Nup62, resulting in the disruption of nucleo-cytoplasmic trafficking, blockage of mRNA export, and impairment of host translation [50,51]. This disruption further results in the cytoplasmic accumulation of SRp20, a cellular splicing factor that acts as an IRES trans-acting factor, binding to the IRES to promote viral translation [50]. EV proteases interfere as well with host transcription. Several studies of PV infection have reported that 3C cleaves transcription factors such as the TATA-binding protein (TBP) [52,53], cyclic AMP-responsive element-binding protein (CREB) [54], octamer binding transcription factor 1 (Oct-1) [55], and p53 [54]. The precursor 3CD also plays an important role in this task. 3CD can enter the nucleus, in which 3C is generated by auto-proteolysis and where transcription factors are accessible [54,56]. Important to note, a catalytically active 2A is required for 3CD nuclear localization as this latter remains in the cytoplasm when expressed alone [56].

The two multitasking proteases also play crucial roles in the escape from the host innate immune response, but their respective involvement is not always clearly established. It is known that 2A and 3C cleave key players in the RIG-I-like receptors (RLRs) signalling pathway. Mukherjee et al. showed that 3C of CVB3 cleaves MAVS [57], while another study by Barral et al. suggests that MDA5 degradation is not mediated by any of the viral proteases but rather occurs in a proteasome and caspase-dependent manner [58]. Later, Feng and colleagues showed that 2A of CVB3, PV, and EV-A71 cleave MDA5 and MAVS, while 3C targets RIG-I [59]. In this study, the authors deny the role of a proteasome and caspase-dependent degradation and suggest that these results are due to the upregulation of MDA5 by poly(I:C) [58]. Furthermore, they mention that although recombinant 2A and 3C proteases can cleave overexpressed MAVS, 3C cleavage products of endogenous MAVS are not observed during CVB3 infection. Overall, this suggests that MAVS is primarily cleaved by 2A and not 3C [59]. Additional studies are still needed to better clarify the respective roles of 2A and 3C in MDA5 and MAVS cleavage. Toll-like receptor (TLR) signalling is also targeted by EVs. 3C of CVB3 and EV-D68 cleave TRIF [57,60], while 3C of CVA16, CVA6, and EV-D68 cleave TAK1 [61], both important adaptors of this antiviral pathway. Another strategy to impair interferon (IFN) production applied by EV-D68 is the cleavage of IRF7 by 3C [62] and the cleavage of TRAF3 by 2A, both key factors for IFN type I production [63]. Finally, 2A proteases of EV-A71, CVB3, CVA21, and EV-D68 seem to prevent the formation of SGs [64]. However, Cheng et al. shows that also 3C of EV-D68 disperses the formation of SG [34]. SGs are associated with IFN-β production and play an important role in cellular innate immunity [64]. Nevertheless, the antagonistic mechanism of EVs remains unclear and further research is needed to clarify the role of 2A and 3C.

In summary, there are multiple examples of EV proteases’ targets. Even though they seem to vary amongst the several EVs, these viruses apply similar strategies to hijack common cellular pathways such as the transcription, translation, and immune response to enhance viral replication.

#### 4.2.2. The Non-Structural Proteins: 2B, 2C, and 2BC

2B is a small hydrophobic protein that localizes to the membranes of the endoplasmic reticulum (ER) and Golgi complex [65,66,67,68]. It is classified as a viroporin. One of the first roles attributed to 2B was the increase in plasma membrane permeability, initially described for PV [69,70,71]. The same was observed for CVB3 and the authors proposed that this increased permeability facilitates the release of viral progeny [72]. Later on, a comparative study evaluating 2B from three different EVs (i.e., CVB3, PV1, and RV-B14) showed that this protein is phylogenetically conserved and presents similar function and subcellular localization [68]. For all three viruses, it reduces ER and Golgi complex Ca^2+^ levels and inhibits protein trafficking [68]. This may contribute to the evasion of the immune response by impairing cytokine secretion, for instance. 2B alterations in Ca^2+^ homeostasis rely on its ability to homo-multimerize and form pores in host membranes, which is supported by several lines of evidence [66,73,74,75,76,77,78]. The subcellular localization of 2B in the ER and Golgi complex supports that 2B increases the permeability of these membranes directly, although the permeability of the plasma membrane is increased by an unknown indirect mechanism [66,67,78].

It is also well known that EVs reorganize ER and Golgi membranes to generate membranous replication organelles, and 2BC is responsible for this phenomenon [73,79]. Even though 2BC is the most active in reorganizing host membranes, its ability to enhance permeability and translocate into membranes resides in its 2B moiety [80]. In addition, expression of 2B in HeLa cells was shown to confer resistance to apoptosis [81]. However, apoptosis is beneficial at the late infection stages as it promotes the egress of virus progeny [82]. Finally, the multifaceted 2B protein seems also to be involved in the induction of autophagy. Autophagy is induced by and promotes the replication of several EVs [83,84,85,86] including EV-D68 [87]. The involvement of 2B in this mechanism is not a surprise considering its involvement in membrane rearrangement. In a nutshell, 2B can form transmembrane pores, disturb Ca^2+^ homeostasis and membrane permeability, and modulate apoptosis and autophagy.

2C is the most conserved protein across EVs [88]. It holds two RNA remodelling activities, an ATP-dependent RNA helicase and an ATP-independent chaperone. Both activities were first described for EV-A71 and CVA16 by Xia et al. [89,90,91]. Knowing that EVs rely on several highly structured cis-acting RNA elements for their replication, the need for such functions comes as no surprise. 2C helicase activity is most likely responsible for unwinding dsRNA, allowing for the recycling of the viral RNA template and promoting the generation of new RNA genomes [89,92]. The 2C chaperone destabilizes and remodels RNA secondary structures such as the 5′ CL and IRES, which is particularly important for RNA replication and translation [89,92]. Inhibition of 2C helicase activity abolished RNA replication and viral production by PV and EV-A71, demonstrating the vital role of 2C in EV replication [89,92]. Moreover, several studies based on mutagenesis experiments and chimeric constructs highlighted specific 2C residues involved in the RNA replication [93], uncoating [94], and encapsidation [93,94,95]. 2C also shares functions with its precursor 2BC. Both present ATPase activity and interact with host intracellular membranes via their amino-terminal region [96]. This interaction allows 2C/2BC to induce the formation of RNA replication complexes [96]. Interestingly, 2C activity goes beyond its role in replication, as it can also contribute to immune evasion. NF-κB is a key player in the innate response as one of the transcription factors responsible for stimulating the expression of IFN, IFN-stimulated genes (ISG), and inflammatory cytokines. 2C prevents NF-κB activation by two different mechanisms: it can either bind and prevent the dimerization of NF-κB or inhibit the phosphorylation of IKKβ and subsequently NF-κB activation [97,98]. In short, 2C seems to be a versatile protein, playing a role in several steps of the viral life cycle (reviewed in Reference [92]).

#### 4.2.3. The Non-Structural Proteins: 3A, 3B, and 3D and Their Precursors

The non-structural protein 3A is a small homodimeric protein that is able to bind membranes via its C-terminal hydrophobic domain [99,100,101]. 3A is also present as its uncleaved precursor 3AB. Interestingly, mature 3A and its precursor 3AB present distinct roles in EV replication [102]. For some EVs such as PV and CVB3 [70,103], 3A was described to inhibit protein trafficking, but this was not the case for other EVs such as EV-A71, RV-A2, and RV-B14 [99,102,104]. For PV and CVB3, this action is accomplished by hampering the function of GBF1 and ARF1 [104,105,106], two host proteins crucial for the assembly and budding of transport vesicles.

Concerning the 3A precursor 3AB, it was observed in the context of PV and EV-A71 infection that this protein exhibits nucleic acid chaperone activity, promoting strand annealing and the destabilisation of secondary structures [102,107,108]. 3AB together with 3CD interacts with both the 5′ CL and 3′ UTR of the positive RNA strand [102,109]. Actually, it has been reported that the full P3 precursor preferentially binds to the 5′ CL, which is subsequently cleaved, releasing 3AB and 3CD to start replication [102,110]. 3AB then promotes the RNA polymerase activity of 3D and stabilizes the complex between 3D, the VPg primer, and the RNA template [102,111,112,113,114,115]. Due to its ability to bind both membranes and 3D polymerase, 3AB acts as an anchor that allows for the assembly of the replication complex [101,102,116].

3B or VPg (virion protein genome linked) is a small protein that serves as primer for RNA replication and remains covalently bound to the genome 5′ UTR [20,102]. 3B is uridylylated by 3D on its third amino acid, a conserved tyrosine, generating VPgpU(pU) and allowing a protein-primed initiation of genome replication [20,102,117]. 3D can use both poly(A) or the *cre* as template for uridylation [27,102]. The precursor 3BC, rather than 3B, might in fact be the substrate for uridylation. This hypothesis is based on evidence showing that 3BC of PV is more efficiently uridylated than the mature VPg [102,118,119] and that, if cleavage of 3BC is inhibited, PV replicons produce viral RNA covalently attached to 3BC [119].

The EV coding sequence ends with 3D, the RNA-dependent RNA polymerase (RdRp). There is no need to emphasize the importance of 3D for viral replication as there are no close homologs in the host cell. The EV RdRp 3D structure resembles a cupped-right hand, with fingers, palm, and thumb subdomains [88,120]. As all primer-dependent RdRps, 3D includes a small thumb subdomain, leaving the active site open and accessible [88]. The catalytic palm is the most conserved subdomain and includes two catalytic aspartic acid residues that coordinate two divalent metal ions essential for 3D activity [88]. The fingers and thumb subdomains interact with each other and are responsible for primer, template, and NTP substrate binding [88].

In essence, the main responsibility of P3 proteins is the replication of the viral RNA and to achieve that, they harmoniously interact with each other and several host factors.

## 5. *Enterovirus D* Receptors

**Figure 4:**
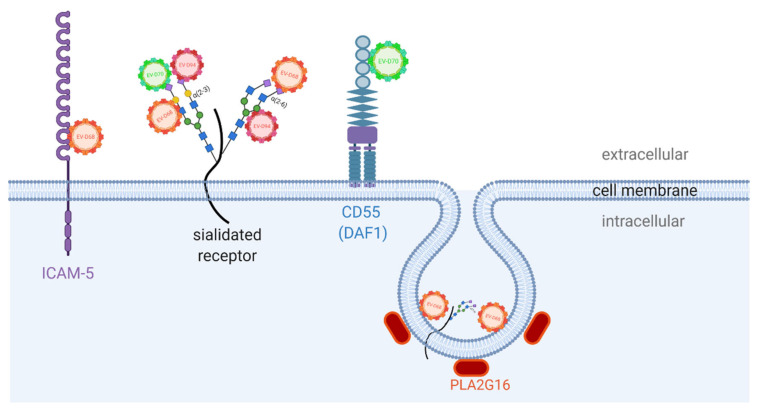
Known binding/attachment receptors identified to date for the Enterovirus D species. EV-D68 was identified to utilize α-2,3 and α-2,6 SAs for attachment and ICAM-5 as a binding receptor. The host factor PLA2G16 aids to initiate the uncoating of EV-D68 into infected cells in a SA-dependent manner. EV-D70 utilizes α-2,3 SAs and DAF1/CD55 as binding and attachment receptors, respectively, while EV-D94 has affinity to α-2,3 SAs and α-2,6 SAs. Receptors for other EVs of this class, namely EV-D111 and EV-D120, have not been identified to date. sGAG are not shown as they may not be true receptors in vivo. Created with BioRender.com, accessed on 15 July 2021.

The availability of cellular receptors is crucial for viral infection and for determining both tissue tropism and the host range. Viruses can sequentially interact with several receptors to infect a cell. Oftentimes attachment factors (such as glycans) are required in order to retain virions at the cell surface. This interaction increases their concentration on the cell surface and consequently increases their likelihood to find the entry receptor, which mediates internalization and the uncoating of the viral particles. EV-D68, EV-D94, and EV-D70 are all known to engage sialic acid (SA) to infect cells (Figure 4), while the receptors employed by the most recently identified EV-Ds, EV-D111, and EV-D120 have not been investigated and remain unidentified [11,12,121].

EV-D70 recognizes α-2,3 linked SA (α-2,3SAs) receptors, while EV-D68 binds to both α-2,3 and α-2,6-linked SA (α-2,3SAs) with a preference for the latter [122,123,124,125,126]. EV-D94 was also reported to bind both α-2,3 and α-2,6SAs (Figure 4) [123].

α-2,6SAs are predominantly found in the upper respiratory tract, specifically on the epithelial cells of the nasal mucosa, trachea, bronchi, and bronchioles. Conversely, α-2,3SAs are more abundantly found in the lower respiratory tract at the level of the alveoli [127]. α-2,3SAs are also abundant in the conjunctival cells of the eye. Understandably, the availability of these glycans correlates with the respiratory and ocular tropism of EV-D68 and EV-D70, respectively. In the gastrointestinal tract, reports have shown a strong presence of α-2,6SAs in the buccal and ileal epithelium, and expression of α-2,3SAs in the epithelium of the colon [128,129,130].

SAs, however, are not the sole receptors of EV-Ds. The decay-accelerating factor (DAF or CD55) was also identified as an attachment receptor for EV-D70 in epithelial cell lines and is reported to be responsible for the atypical tropism of this virus to the eye conjunctiva (Figure 4) [131,132]. Interaction with DAF is, however, not exclusive for EV-D70, having been identified as the cellular receptor for several echovirus types [133], CVBs [134,135,136] and CVA21 [134,135,136]. In spite of this, other uncharacterized receptors might interact with EV-D70 as it is able to infect leukocyte cell lines that express little to no DAF on their surface [137]. While the attachment of CVBs to DAF activates signalling in epithelial cells to expose an uncoating receptor, namely the coxackievirus-adenovirus receptor (CAR) from tight junctions [138], the same has not been described for EV-D70.

More focus has been given to studying the receptor engagement by EV-D68 over the past decade. ICAM-5, a surface receptor enriched in telencephalic grey matter, has been proposed as an EV-D68 receptor (Figure 4) [139]. It was postulated that the interaction with this molecule could account for the virus neural tropism and the association with acute flaccid myelitis (AFM) [5]. Nevertheless, it has been shown that EV-D68 has the ability to infect neurons and astrocytes in an ICAM-5 and SA-independent manner [140,141]; thus, the role of ICAM-5 in EV-D68 neurotropism is still debated. In addition to ICAM-5, a contemporary EV-D68 strain, EV-D68-947, was found to engage with sulfated glycosaminoglycans (sGAGs) and generate a productive infection in SA-deficient cell lines [142]. Interestingly, the interaction with sGAGs enables EV-D68 to bypass a common pan-EV uncoating host factor, PLA2G16, (Figure 4) indicating that the receptors engaged by the different strains of the virus can result in alternative infection pathways [142,143,144]. However, this engagement could be a result of cell line adaptation and sGAG might not represent a true receptor in vivo [144].

The binding of SAs or sGAGs to the canyon regions of the virion releases the pocket factor, which destabilizes the capsid proteins and consequently initiates the uncoating of the viral genome into the cell cytosol [125,142,145]. There is increasing evidence that EV-D68 might not require a single specific protein receptor to interact with and initiate viral entry. In fact, the binding to SAs on the VP1 GH loop of the canyon region, in synergy with endosomal pH acidification, have been identified as the main cues for the uncoating and viral genome release [37,125,142,145]. Once inside the endosomal compartment, pH acidification induces a series of conformational changes on the native virion, which expands (forming pores on the capsid-E particles), converts into A particles (devoid of VP4 proteins and externalized VP1 N-terminus), and the viral genome is then released from these pores [146]. PLA2G16 then aids the viral release from the endosome to the cytosol by inducing the pore formation of the endosomal membranes [143]. Therefore, the diversity of receptors used by EV-D68 identified to date could suggest a dual-receptor system usage characterized by glycan engagement and protein/host factor interactions [142].

The ability of EV-D members to employ a large range of receptors contributes to their diverse tropism and contributes to causing such diverse pathologies. In addition, while EV-D111 from human and simian samples are not phylogenetically distinct [11] and simian EVs seem to be able of cross-species transmission [12], it is unclear if EV-D receptors contribute to the species barrier and whether prior viral adaptation is needed to jump from non-human primates (NHP) to humans. Such knowledge is critical to understand the zoonotic potential of EV-Ds or of other animal EVs. Finally, the current understanding on the receptors utilized by the members of *Enterovirus D* species is still limited. This proves just how complex and versatile EVs can be and that further debate and studies are needed. The identification of the binding/attachment receptors is also crucial for the development of novel antiviral strategies and for the establishment of more coherent study models in the laboratory setting.

## 6. *Enterovirus D* Pathogenesis and Associated Symptoms

**Figure 5:**
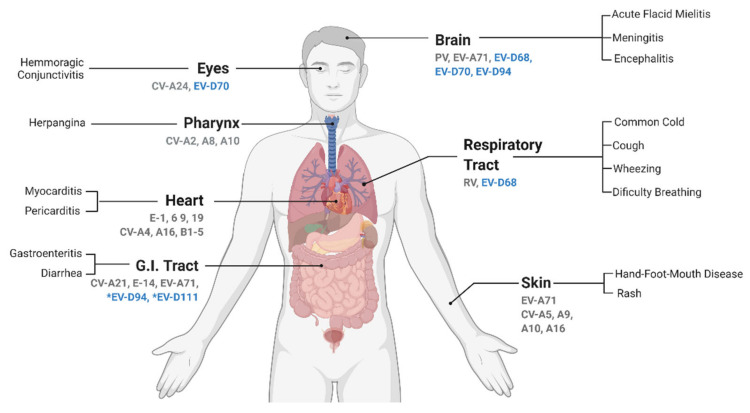
Tropism and associated symptoms of human enterovirus infections. The most commonly reported symptoms associated to each EV infection in the human host are depicted, grouped by organ. Highlighted in blue are the members of the Enterovirus D species. All EV-Ds infecting humans have been associated with neural infections, although a causal link remains to be demonstrated. Their primary replication site is reported to be initiated either in the eye (EV-D70), respiratory tract (EV-D68), or gut (EV-D94 and EV-D111). Even though there is an association between *viruses found in stools, no gastro-intestinal symptoms are reported. Abbreviations: EV, enterovirus; RV, rhinovirus; CV, coxsackievirus; PV, poliovirus; and E, echovirus. Adapted from: “Expression of ACE2 Receptor in Human Host Tissues” by BioRender.com (2021). Retrieved from https://app.biorender.com/biorender-templates, accessed on 15 July 2021.

EVs are well known for causing a plethora of symptoms (Figure 5). Even though most infections are commonly asymptomatic, they can lead to life-threatening complications, predominantly in children of young ages, immunocompromised adults, or adults with other underlying morbidities [3,147,148,149]. The vast majority of EVs are transmitted via the fecal–oral route and/or contact with respiratory secretions. Their primary infection sites are typically the mucosal surfaces and normally the infection is restricted to the primary infection site [150,151]. However, infection can spread and evolve into a viremic phase, disseminating to other tissues via lymphatic or blood circulation. In more severe outcomes, the infection can reach the central nervous system (CNS), which can result in meningitis, encephalitis, paralysis, and possibly death (Figure 5) [151,152].

EV-D70 has a rather distinctive disease association and it has been identified as the cause of several outbreaks of acute hemorrhagic conjunctivitis worldwide. It was first isolated in 1971 after an initial outbreak in Ghana in 1969, which spread to other parts of the world in the following years (South-East Asia, Japan, India, and England) [6,153]. Common symptoms of the disease include eye pain, swelling of the eyelids, redness of the conjunctiva, excessive tearing, photophobia, and eye discharge [154]. The symptoms usually subside without the need for treatment in about 10 days. Typically, EV-D70 reaches the conjunctival and corneal epithelium by hand-to-eye transmission of the virus from infected surfaces or objects [154]. It is a cytopathic virus that induces cell apoptosis [155] and the shedding of cells into the eye secretions correlates with the symptomatology of conjunctivitis. EV-D70 is also able to infect several lineages of leukocytes [137], which are also abundantly found in eye discharges. It is through these discharges (and poor hygiene conditions) that the virus spreads to other people [154]. Despite being rare, hemorrhagic conjunctivitis caused by EV-D70 can also cause non-ophthalmic symptoms such as neuronal dysfunction [154,156]. The capacity of EV-D70 to infect leukocytes could be the mechanism by which it reaches the CNS via lymphatic circulation [137]. Nevertheless, this hypothesis was not yet elucidated.

Enterotropic EV-D94 and EV-D111 have been isolated from stool samples of humans in African countries [10,11,157], while EV-D120 has only been identified in non-human primate feces [12]. No exact symptomatology has been described for EV-D120 and EV-D111 and even less is known about their pathogenesis. Due to its origin, its acid-resistance and optimal growth at 37 °C, EV-D94 has been postulated to cause enteric disease and to be transmitted via the fecal–oral route [10]. The primary target tissue of EV-D94 is currently not known but in vitro experiments reveal that EV-D94 is able to infect leukocytes, endothelial cells, and human pancreatic islets [150]. EV-D94 may also cause neurologic symptoms given that it was first isolated from a child from the Democratic Republic of Congo who was suffering from AFP [157].

The respiratory member of the *Enterovirus D* species, EV-D68, is likely the most popular as it has been the cause of biennial outbreaks in the pediatric population in recent years. However, this is not a recent virus but rather a re-emergent one. It was first isolated in California in 1962 from children with pneumonia and bronchitis (giving rise to the Fermon, Rhyne, Franklin, and Robinson strains) [158]. It is acid liable and has an optimal growth at 33 °C, as opposed to its enteric counterparts. Due to its similarity with other RVs, it was first classified as human rhinovirus 87 and only later re-classified as EV-D68 [159]. Its clinical presentation can vary from no symptoms or mild respiratory disease (runny nose, sneezing, cough, and body and muscle aches) to severe respiratory pathology (with wheezing, difficulty of breathing, and fever) or even life-threatening neurologic syndromes [5,148]. During a widespread outbreak in the United States of America in 2014, EV-D68 gained public interest due to its high morbidity in children [148]. Severe complications arose from respiratory illness to a polio-like disease, described as AFM [160]. The recently characterized strains of EV-D68 from the 2014 outbreak seem to be less susceptible to attenuation at 37 °C, can infect neural tissues independently of SA, and can utilize the neural receptor ICAM-5 (abundantly found in the telencephalon and also identified as a receptor for prototypic strains) [123,161,162,163]. Infection via intramuscular injection in neonatal mice using several contemporary strains of this virus results in limb paralysis and interestingly the severity of the paralysis is age-dependent [164]. A follow-up study from the same group suggests that the virus reaches the CNS by retrograde axonal transport via neuromuscular junctions, from the distal axons of motor neurons and spreading up to their neuronal cell bodies [140].

## 7. Conclusions

In this review, we have summarized the current knowledge on EV-D members and the known strategies used by EVs to hijack the host cell machinery. Despite its small size, the *Enterovirus D* species contains relevant human pathogens able to cause a wide-range of symptoms including respiratory illness, eyes infections, and neurological complications. However, there is still a lot to learn about this peculiar EV species. There are currently no vaccines or antivirals available against EV-D members. Since the 2014 outbreak, EV-D68 has become the most popular and feared EV-D due to its increased virulence, associated with severe respiratory disease and novel neurotropic potential. As a result, most antiviral research has targeted this virus. Several antiviral strategies have been proposed (reviewed in References [165,166]) such as capsid-binding compounds [145,167,168,169,170,171], viral proteases’ inhibitors [168,169,172,173,174], drugs targeting the non-structural proteins 2C [168,175,176], 3A [167,168,169,177], the viral polymerase 3D [167,169,178,179] and drugs blocking translation [180]. However, even the most favourable candidates did not obtain promising results in the mouse models [175,181].

In addition to the possible re-emergence of EV-D members already established in the human population with new virulence factors, the existence of a large animal reservoir poses the threat of the zoonotic emergence of new neurotropic variants. Phylogenetic analysis of EV-D111 strains suggests recent zoonotic transmission between NHP and humans [11], consistent with the zoonotic origin proposed for EV-D70 [182,183]. Similar observations have been made for other EVs [121,184]. Surveillance of EV-Ds or other EVs circulating in NHP is thus critical to be prepared in case of the emergence of novel variants in an immunologically naïve population.

To conclude, further studies on EV-Ds are needed not only to better understand the wide range of diseases caused by these viruses in humans and to find effective antiviral strategies, but also to better apprehend the zoonotic potential of these viruses.

## Supplementary Materials

The following are available online at https://www.mdpi.com/article/10.3390/microorganisms9081758/s1, Figure S1: Sequence and alignment of the cis-acting elements present in EV-D genomes.
