# Supplementary material for: Enterovirus D: A Small but Versatile Species
Source: Microorganisms. 2021 Aug 17;9(8):1758. doi: 10.3390/microorganisms9081758 (PMC8400195; doi:10.3390/microorganisms9081758)
Supplement: Supplementary file 1 [file microorganisms-09-01758-s001.zip › microorganisms-1325600-supplementary.pdf]

microorganisms

Supplementary Materials

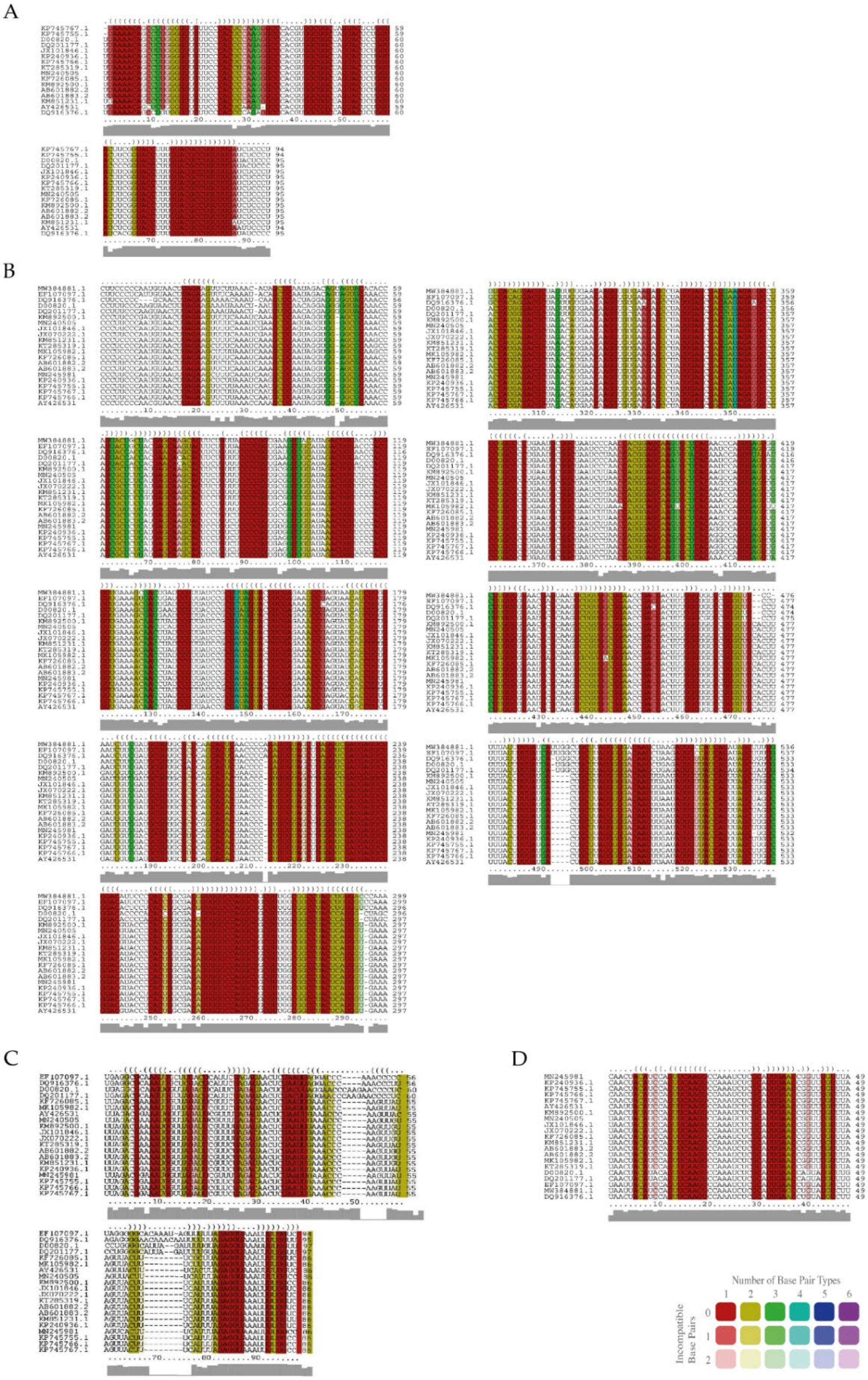

Figure S1. Sequence and alignment of the cis-acting elements present in EV-D genomes. 5′ CL (**A**), 5′ IRES (**B**), 3′ UTR (**C**) and cre (**D**). Sequences are identified by their respective genebank accession number: EV-D68 (AY426531, AB601882.2, AB601883.2, JX070222.1, JX10184.1, KF726085, KM851231.1, KM892500.1, KP240936, KP745755.1, KP745766.1, KP745767.1, KT285319.1, MK105982.1, MN240505, MN245981), EV-D94 (DQ916376, EF107097), EV-D70 (DQ201177, D00820) and EV-D111 (MW384881), incomplete sequences were excluded. The RNA fragments were extracted using AliView [18] sequence alignment editor and then were simultaneously folded and aligned using LocARNA from Freiburg RNA tools, the respective structures are in Figure 2.
